# Optimization and Characterization of Khellin Loaded Nanogels for the Potential Use in Psoriasis Management

**DOI:** 10.2174/0113892010375509250429052937

**Published:** 2025-05-05

**Authors:** Sehernaz Topuzoğlu, Evren Algın Yapar, Lüceyn Abdo, İmren Esentürk-Güzel, Aslı Gürbüz Yurtsever, Selin Gümrükçü, Meryem Sedef Erdal, Rakesh K. Sindhu

**Affiliations:** 1 Department of Pharmaceutical Technology, Hamidiye Faculty of Pharmacy, University of Health Sciences, Istanbul, Türkiye;; 2 Department of Pharmaceutical Technology, Faculty of Pharmacy, Sivas Cumhuriyet University, Sivas, Türkiye;; 3 Department of Pharmaceutical Technology, Faculty of Pharmacy, Istanbul University, Istanbul, Türkiye;; 4 Department of Chemistry, Istanbul Technical University, Istanbul, Türkiye;; 5 Department of Pharmaceutical Sciences, Guru Jambheshwar University of Science and Technology, Hisar 125001, Haryana, India

**Keywords:** Ammi visnaga L., khellin, psoriasis, nanogel, topical, drug delivery

## Abstract

**Background:**

Psoriasis is a chronic skin disease that affects patients' quality of life. Treating psoriasis remains a significant challenge due to various factors, including individual response variability, drug resistance, and the range of side effects associated with currently available medications. Nowadays, numerous research efforts are being made aiming at overcoming the obstacles of the available psoriasis treatments are still taking place.

**Objectives:**

This research aims to develop and evaluate a nanogel formulation loaded with khellin for the effective treatment of psoriasis.

**Methods:**

Khellin nanogel was prepared using the self-assembly method with a synthesized gelatin-pluronic copolymer. The novel formulation was characterized *via* size, size distribution, encapsulation efficiency, *in vitro* release, and* ex vivo* skin deposition.

**Results:**

The final nanogel formulation had an average size of 119.6 nm, a polydispersity index of 0.248 and an excellent encapsulation efficiency of 88%. *In vitro* drug release study demonstrated that nanogels showed a great accelerated drug release profile by releasing khellin within 2 hours, which is suitable for photochemotherapy applications. In addition, khellin-loaded nanogel formulation had 1.7 times better skin deposition potential than the control gel formulation.

**Conclusion:**

The prepared nanogel formulation provides a high potential to be an ideal drug delivery system of khellin in combination with phototherapy for more efficient psoriasis treatment.

## 1 INTRODUCTION

Psoriasis is a common, chronic, and non-contagious skin disease of an unknown cause and treatment, which adversely affects the quality of life. This disease is characterized by the formation of red, scaly, raised plaques as a result of epidermal hyperproliferation and abnormal keratinocyte differentiation [1-3]. Besides these physiological effects, it is reported that depression and anxiety disorders are frequently encountered in psoriasis patients due to the cosmetically unpleasant appearance of these plaques on the skin. Moreover, stress triggers psoriasis as well [4-6].

Topical therapy is the most common treatment route for psoriasis because the majority of patients (70-80%) are diagnosed with localized plaques. In addition, topical therapy could be used in combination with phototherapy or systemic treatments for patients with resistant psoriatic lesions and plaques. Therefore, treatment with effective and safe topical drugs that directly target the lesion has great importance [1]. In the past years, mainly coal tar, dithranol, and salicylic acid have been used in the treatment of psoriasis. Today, first-line drugs are accepted as corticosteroids, vitamin D analogues, tazarotene, anthralin, tacrolimus, and pimecrolimus [1, 4]. Phototherapy is commonly employed in the treatment of resistant psoriatic lesions and plaques, utilizing ultraviolet A (UVA) or ultraviolet B (UVB) light in combination with various topical agents. For instance, psoralen—a photosensitizing agent—is often administered alongside UVA light (PUVA therapy) for managing psoriasis [7].

Phototherapy acts as a complementary and alternative therapy for diminishing psoriasis symptoms [8-10]. *Ammi visnaga* L. grows naturally in regions with a Mediterranean climate, containing γ-pyrones (such as khellin), coumarins, and flavonoids, whereas, khellin is one of the major components of this plant [11, 12]. Khellin is a furanochrome substance extracted from *Ammi visnaga* seeds, and it is used in photochemotherapy of skin diseases. When exposed to UV radiation (365 nm), khellin shows its effect by forming a molecular complex with DNA [13, 14]. In a study conducted in 1983, the impact of orally administered khellin on psoriasis was investigated in 10 patients. After 4 months of oral administration of khellin and sunlight, positive results were observed in 8 patients [15]. However, oral administration of khellin has side effects such as nausea, elevation of liver enzymes, and the risk of hepatotoxicity. In topical application, these systemic side effects are not observed. In addition, since the substance does not undergo first-pass metabolism, it will have a higher effect compared to systemic application [16, 17]. However, since khellin has low solubility in water (0.025% w/v), there is a need to develop innovative methods and systems that improve release properties and drug penetration into the skin [18-20].

Drug delivery system release profiles must be carefully tailored to align with the active molecule's mechanism of action. According to the literature, when khellin is applied to the psoriatic region and exposed to UV radiation, its aromatic structure generates reactive oxygen species, causing DNA and cell membrane damage in keratinocytes. One hour after the damage occurs, DNA repair and cell proliferation are initiated, reversing the damage within 48 to 72 hours, resulting in the healing of psoriatic lesions during this period [21-23]. Building on this premise, it is crucial to ensure that a sufficient concentration of khellin molecules reaches the target region before UVA exposure, to maximize temporal effects before the onset of the healing stage. Accordingly, this study aims to design a drug delivery system with enhanced skin penetration and a rapid drug release profile (Fig. **1**).

Nanogels are nanosized hydrogel systems, typically exhibiting a spherical shape and characterized by a three-dimensional, nanoscale structure formed of swellable polymer networks. These networks are cross-linked either chemically or physically, allowing nanogels to absorb and retain large amounts of water while preserving their structural integrity [24].

Due to its high flexibility, biocompatibility, and stability, nanogel has a long residence time on the skin, can easily penetrate deep tissues, and has a high drug-carrying capacity. Moreover, with the use of nanogels, controlled and targeted drug release could be performed, which would significantly reduce the side effects of drugs and increase their therapeutic efficacy [25-27]. Gelatin is a natural, biocompatible, and biodegradable polymer obtained upon hydrolysis of collagen, one of the basic protein building blocks of the skin. It is frequently used in the development of nanocarrier systems in the pharmaceutical and cosmetic fields because of its superior properties [28-30]. However, due to the lipophilic nature of khellin, it is difficult to load it into nanogels consisting of gelatin only. Therefore, it will be easier to load khellin into nanogels if gelatin is conjugated with the amphiphilic pluronic polymer [31-36], which is a triblock copolymer, consisting of hydrophobic [poly (propylene oxide)] and hydrophilic (poly (ethylene oxide)) chains. A nanogel is created in the aqueous medium when the hydrophobic chains are encircled by the hydrophilic chains of the polymer [37-40]. It is expected that the nanogel formulations, prepared in this way, can release khellin rapidly and deliver it to the lower layers of the skin as desired.

In this study, a topical nanogel formulation of khellin to be used against psoriasis was developed. Khellin was loaded into nanogels made of pluronic and gelatin co-polymer. Size, size distribution, and encapsulation efficiency of the nanogel formulations were characterized besides drug release and skin deposition studies [41-50].

## 2 MATERIALS AND METHODS

### 2.1 Materials

Khellin (CAS: 82-02-0), Pluronic P123 (Mw 5.800), p-nitrophenyl chloroformate (NPC) (CAS: 4264-83-9), 3-amino-1-propanol (Ami), gelatin (CAS: 9000-70-8), and cellulose membrane (12.000-14.000 Mw) were purchased from Sigma-Aldrich Chemie Gmbh (Steinheim, Germany). Diethyl ether, hexane, chloroform, acetone, ethanol, and methanol were supplied from ISOLAB (Istanbul, Türkiye). Pig skins were obtained from Tropical Pig Farm (Antalya, Türkiye). All other reagents used were of analytical grade.

### 2.2 Synthesis of Gelatin-pluronic P123 Copolymer

Gelatin-Pluronic copolymer was synthesized using the following method stated in the literature [41, 42]. Firstly, gelatin was dissolved in distilled water at 40 °C, stirred for 1 hour, and stored below 25°C. Later on, previously modified NPC-p123 was dissolved in water at 4 °C and added dropwise to the gelatin solution under continuous stirring at 25 °C for 24 hours. The reaction mixture was dialyzed against cold deionized water using a cellulose membrane for 3 days, followed by lyophilization to obtain pluronic-conjugated gelatin in solid form.

### 2.3 Characterization of the Synthesized Gelatin-pluronic Copolymer

Synthesized Gelatin-Pluronic P123 copolymer's chemical structure was characterized using Proton Nuclear Magnetic Resonance (1H-NMR) spectrometers. NMR spectra were recorded at room temperature, with deuterium oxide (D_2_O) as a solvent and trace of water as an internal reference [43].

### 2.4 Nanogel Preparation

Nanogel was prepared by modifying the "self-assembled" method registered in the literature [42]. Preformulation studies parameters are given in Table **1**. To prepare nanogel formulations, the amphiphilic Gelatin-Pluronic copolymer was first dissolved in distilled water. Subsequently, the organic phase was prepared by dissolving a precise amount of khellin in the organic solvent (acetone, ethanol or methanol). The khellin solution was then added dropwise to the Gelatin-Pluronic copolymer solution at a rate of 4 ml/h while continuously mixing at 250 rpm at room temperature. The organic solvent was subsequently evaporated from the mixture in a fume hood (Fig. **2**).

### 2.5 Nanogel Characterization

The size of the nanogels and the zeta potential of the formulation were both measured using a Malvern Zetasizer (Malvern, UK). An analysis of the size was conducted via the dynamic light scattering method, and the polydispersity index (PDI) was measured to assess the uniformity of the nanogel size [44].

### 2.6 Determination of Encapsulation Efficiency

To determine the amount of khellin loaded in the nanogels, a method based on centrifugation for leaching of khellin from nanogels and then its quantification by analyzing with High Performance Liquid Chromatography (HPLC) was used, also formerly utilized and indicated in our previous study [45, 46]. The following equation (Eq. 1) was used to calculate the encapsulation efficiency:

Encapsulation efficiency=Loaded active substance/Total amount of active substance x 100 % (Eq. 1)

### 2.7 In vitro Drug Release

Franz diffusion cells (Permegear V6A Stirrer, Hellertown, USA) were used to evaluate *in vitro* khellin release from the formulations. A method based on Franz diffusion cells for khellin release and its analysis with HPLC was used [45, 47].

### 2.8 Ex-vivo Skin Deposition Study

Franz diffusion cells (Permegear V6A Stirrer, Hellertown, USA) were used to evaluate the *ex vivo* skin deposition property of nanogels. The diffusion cell was assembled with pig back skin separating the donor from the receptor compartment. Pig skins were obtained from a local slaughterhouse (Ethical approval is not required).

### 2.9 Attenuated Total Reflectance-Fourier Transform Infrared (ATR-FTIR)

To analyze the penetration of khellin through the skin, alterations in the skin's lipid structure were evaluated. The molecular sequence of the lipid structure in the stratum corneum can be defined using ATR-FTIR spectroscopy [49]. The carbon-hydrogen (C-H) symmetric (2850 cm^-1^) and asymmetric (2920 cm^-1^) stretching bands' wave numbers move to greater values as a result of the formulation components' interaction with the alkyl chains of the intercellular lipids in the stratum corneum [50]. To determine whether these interactions successfully occurred or not, skin samples used in the skin deposition study were tested using ATR-FTIR spectroscopy analysis. The formulation and receptor phase remnants were gently wiped from the skin's surface using a napkin. Subsequently, skin samples were positioned with the stratum corneum surface facing the crystal on an ATR-FTIR (Nicolet^TM^ 6700 Continuum^TM^ Infrared Microscope, Thermo Fisher Scientific, Switzerland) tray. To guarantee sufficient contact between the crystal and the skin

sample, a steady force of 60 N was consistently applied to the skin. Analysis was carried out with a spectral resolution of 4 cm^-1^, covering the frequency range of 4000-550 cm^-1^ [50, 51].

### 2.10 HPLC Analysis

HPLC analysis was adapted from the methodology described in the literature [52], and the specific parameters for the HPLC setup, including the system components, mobile phase, and operating conditions, are provided in Table **2**.

### 2.11 Stability

Formulations were kept at 2-8 °C or 25 ˚C - %60 relative humidity storage conditions. Using the "dynamic light scattering" method (Malvern Zetasizer, UK), the samples' size and PDI values were assessed at regular intervals for a period of 20 weeks [53-56].

## 3 RESULTS

### 3.1 Synthesis of Gelatin-pluronic Copolymer and Characterization by 1H-NMR

Gelatin-Pluronic copolymer was synthesized as described in the materials and methods section. ^1^H-NMR spectra were compared to another study, and identical results were obtained [44]. The ^1^H-NMR spectra of the samples exhibited chemical shifts in D_2_O. Fig. (**3a**) shows the ^1^H-NMR spectrum of the structure. The resonance peaks at 7.40 ppm and 8.29 ppm show the aromatic protons in the structure. The peaks at 1.23 ppm are assigned to -CH_3_ protons, and the peaks at -3.82 ppm are for -CH_2_. The peak at 4.45 ppm indicates formation of NPC-CH_2_ moieties in activated P123.

Fig. (**3b**) shows the ^1^H-NMR spectrum of the NPC-P123-OH compound. The existence of a new peak at ~4.22 ppm corresponds to the methylene group bound to the Ami moiety. This shows that NPC-P123-OH was successfully synthesized. Fig. (**3c**) shows the ^1^H-NMR spectrum of the successfully synthesized Gelatin-Pluronic copolymer.

In addition, it was determined that the formulation with the best PDI value among all used organic phases was acetone (Table **3**).

### 3.2 Preparation and Characterization of Nanogels

#### 3.2.1 Effects of the Type and Evaporation Method of the Organic Phase

In the preformulation studies, the organic phase effect was evaluated. Methanol, ethanol, and acetone were used as organic phases. In the dynamic light scattering results, it was observed that the size of the nanogels was larger in formulations prepared with methanol and ethanol compared to those prepared with acetone. Moreover, more than one peak was obtained in the size distribution graphs of methanol and ethanol. On the other hand, formulations prepared with acetone had a single peak.

An unheated and heated rotary evaporator, and fumehood methods were tested to determine the most suitable organic phase evaporation method. It was found that the structure deteriorates with the increase of heat (Table **4**). Then, the fumehood method and unheated rotary evaporator methods were compared, and similar results were obtained (Table **3**). Therefore, since evaporation with a fume hood is more practical, this method was preferred.

#### 3.2.2 Effect of Polymer Concentration on Size and Encapsulation

The polymer concentration effect on size and encapsulation efficiency was compared using 0.7%, 1.4%, and 2.1% (w/v) polymer solutions. Formulation prepared with 1.4% polymer concentration was found to have the smallest nanogel size and the best encapsulation efficiency (98%). On the other hand, the nanogel capacity of 0.7% polymer concentration was insufficient to load the khellin molecules. Therefore, the nanogels’ size increased significantly, and in this sample, the encapsulation percentage decreased to 54%. In addition, precipitates and a very blurry appearance were obtained in this formulation (Fig. **4**). Nanogels made with higher polymer concentration (2.1%) had a high encapsulation efficiency; however, a slight increase in size was also observed (Fig. **5**).

#### 3.2.3 The Effect of the Amount of Active Substance on Size and Encapsulation

To determine the effect of the amount of active substance on size and encapsulation efficiency, formulations containing 0.5 mg, 1 mg, 2.5 mg, 5 mg, and 10 mg khellin were tested. The encapsulation results of formulations containing 0.5 mg, 1 mg, and 2.5 mg khellin were found to be over 95%. In these formulations, a slight increase in the size of the nanogel was observed as the amount of khellin increased (Fig. **6**). However, in formulations containing 5 mg and 10 mg of khellin, an important decrease in encapsulation values, a significant increase in particle sizes and precipitates in the formulation were observed. In addition, irregular peaks and high PDI values were obtained in the size distribution graph. Accordingly, it was found that the formulation in which the highest amount of active substance could be loaded without forming precipitates and with a high encapsulation efficiency of 98% was the 2.5 mg formulation.

#### 3.2.4 Effect of Homogenization and Sonication

Application of homogenization and sonication to the formulations at the last stage during the preparation of the nanogels did not provide an improved effect but also caused deterioration of the nanogel structure (Table **5**). With the application of homogenization and sonication, higher PDI values and irregular size distribution graphs were obtained.

### 3.3 Preparation and Characterization of the Final Formulation

Based on the preformulation results, the final formulation was prepared using the following optimized parameters: 1.4% of the amphiphilic Gelatin-Pluronic copolymer was dissolved in 7 mL of distilled water. Simultaneously, the organic phase was created by dissolving 2.5 mg of khellin in 2 mL of acetone. The khellin solution was then gradually added to the Gelatin-Pluronic copolymer solution at a rate of 4 mL/h, while mixing at 250 rpm at room temperature. After combining the solutions, the organic solvent evaporated under an unheated fume hood. This formulation exhibited an average particle size of 119.6 nm, a polydispersity index (PDI) of 0.248, and a zeta potential of -4.103 mV. The encapsulation efficiency of the formulation was determined to be 88%, indicating excellent encapsulation.

### 3.4 In vitro Drug Release Study

In the *in vitro* release study, it was observed that the nanogel showed an excellent accelerated release. As described above, rapid release is important for the mechanism of action of khellin. The nanogel showed a much faster release compared to the control gel formulation (Fig. **7**). It was observed that 86% of loaded khellin was released during the first hour and almost all of the loaded active substance was released within 2 hours, while the control gel released around 60% of the loaded amount even after 24 hours.

### 3.5 Ex-vivo Skin Deposition Study

The results of the skin deposition study showed that the skin accumulation of khellin applied with the nanogel formulation is 20.830 ± 2.460 μg/cm^2^ while the accumulation is 12.415 ± 2.316 μg/cm^2^ when applied in control gel.

#### 3.5.1 Fourier Transform Infrared Spectroscopy (FTIR)

ATR-FTIR spectra taken from the analysis performed as described in Method 2.2.5. The changes in wave numbers of carbon-hydrogen (C-H) symmetric and asymmetric bands were 2850 cm^-1^ and 2920 cm^-1^ respectively.

### 3.6 Stability of Nanogels

Results of the stability of the nanogel formulations of 2 - 8 °C and 25±2˚C/%60±5 storage conditions are given in Table **6**. As seen from the results, the formulation was stable in both conditions.

## 4 DISCUSSION

In this study, the potential of using GP copolymer nanogel as a khellin carrier for psoriasis treatment was examined. In this regard, GP copolymer was successfully synthesized and used to form nano-sized gel structure. To optimize the nanogel properties, the method parameters were evaluated individually, and the best parameters were selected for the final formulation.

The effect of different organic phase solvents on formulation properties was investigated. Methanol, ethanol, and acetone were used as organic phases. According to the obtained result, it was observed that the organic phase with the best results was acetone. This could be explained by the polarity of the organic solvents. Previous studies also showed that smaller particle sizes and narrower size distribution were obtained when less polar organic solvents were used [57-59].

For organic phase evaporation, heated and unheated rotary evaporator methods, and the fume hood method were tested. It was found that applying heat increased the distribution of particle size significantly. It was found that applying heat significantly increased both the particle size and its distribution. This observation is consistent with other studies in the literature, which also demonstrate that increasing temperature leads to an increase in particle size and a broader particle size distribution [60, 61]. This shows that heat application deteriorates the GP nanogel structure. However, the unheated rotary evaporator and fume hood method gave similar results in both drug-unloaded and drug-loaded formulations.

Polymer concentration effects were evaluated using different polymer solutions and constant amounts of active substances. It was found that particle size increases with the increase in polymer concentration, which aligned with the findings reported in the literature [62-64]. However, inadequate polymer concentration was found to be disadvantageous as well. Low polymer concentration caused the drug loading capacity of nanogels to be exceeded. This led to a significant decrease in the encapsulation efficiency and an increase in nanogel size. For this, polymer concentration was best optimized to 1.4% with the best loading capacity and particle size. Keeping the polymer concentration constant, the effects of the amount of the active substance on the size and encapsulation efficiency were also tested. A slight increase in the size of nanogels was observed as the amount of khellin increased. However, excessive amounts of khellin led to a significant drop in encapsulation efficiency, an important increase in particle size, and the formation of precipitates. Furthermore, irregular peaks and high PDI values were obtained in the size distribution graphics of these formulations. This can be explained by the nanogel's loading capacity being exceeded, and similar situations were also mentioned in the literature [65-70]. Effects of polymer concentration and the active substance amount on particle size and encapsulation efficiency were related to the polymer-to-drug ratio. This ratio has a limiting value at which no drug molecule can be encapsulated. Precipitate formation and causing reduced drug load efficiency occur when this ratio was exceeded [71].

The utilization of homogenization and sonication techniques in the final stage of nanogel preparation resulted in the degradation of the nanogel structure. This phenomenon can be attributed to the heat generated during these processes, as it was previously discussed that heat caused the structure to deteriorate.

After this series of preformulation studies, the most optimized final nanogel formulation was selected. The final nanogel formulation had an excellent encapsulation efficiency of 88%, a 0.248 PDI value, and an average particle size of 119.6 nm. Since this formulation had the smallest particle size with fairly acceptable PDI values, it was chosen as the final optimized formulation, even though the encapsulation efficiency as high as 98% was achieved in other formulations. The small-sized nanogel formulation was chosen as it was expected to increase skin penetration in clinical applications [72-74].

*In vitro* release study findings revealed that our nanogel formulation exhibited a significantly accelerated drug release profile. 86% of the loaded khellin was released during the first hour, and almost the entire amount of khellin loaded to the nanogel was released within 2 hours, while the control gel released only around 60% of the loaded amount even after 24 hours. This accelerated release profile is very suitable for the mechanism of khellin. Khellin was expected to cause short-term DNA and cell membrane damage after UVA application. 1 hour after this effect, the damage began to reverse within 48 - 72 hours, and the psoriatic lesions healed [10]. The obtained rapid release is expected to enable khellin molecules to reach the target area quickly so that maximum efficiency could be achieved when UVA radiation is subsequently applied. Besides this, the fact that the formulation released no more khellin after 2 hours was expected to ensure that the healing process was not interrupted by further delayed release of khellin, which could potentially cause unwanted short-term DNA and cell membrane damage.

*Ex-vivo* skin deposition study results showed that the khellin-loaded to nanogel formulation had 1.7 times better skin deposition than khellin in the control gel, which suggests that the nanogel grants improved skin permeation. It is well known that nano-sized carrier systems have a strong skin deposition ability, so they have more potential for topical applications [72, 73].

Results of the stability of the nanogel formulations at 2 - 8 °C and 25±2˚C/%60±5 storage conditions were evaluated, and it was found that the formulation is stable in both conditions. It is well known that nanogels increase the stability of loaded active substances [75, 76]. Khellin is a photoactive molecule in the furanchromone structure. For this, degradation and instability in the molecule could be seen during the storage process [77]. Our stable nanogel formulation was expected to have increased khellin stability for prolonged storage duration.

As a result, Gelatin-Pluronic copolymer was synthesized successfully and was used in the preparation of the self-assembled nanogel formulation. The final nanogel formulation, which was chosen after a series of preformulation studies, had an average size of 119.6 nm, a PDI of 0.248, and an excellent encapsulation efficiency of 88%. In-vitro release study demonstrated that the nanogels showed a great accelerated drug release profile. Almost all khellin-loaded into the nanogel was released within 2 hours, while the control gel released only around 60% of the loaded amount even after 24 hours. This rapid release profile is very suitable for the mechanism of khellin. The formulation released khellin according to the Korsmeyer-Peppas model with a calculated 'n' number of 0.058. This indicates that the formulation shows a controlled release. *Ex vivo* skin deposition study demonstrated that khellin in the nanogel formulation had 1.7 times better skin deposition than khellin in the control gel. Regarding these results, our nanogel formulation was found to be a promising formulation for khellin delivery, offering great potential for enhanced psoriasis treatment when combined with phototherapy.

## CONCLUSION

In this research, a khellin-loaded nanogel formulation was successfully developed for potential use in psoriasis treatment. Gelatin-Pluronic copolymer was synthesized and utilized in the preparation of the self-assembled nanogel formulation. The final formulation was selected based on a series of pre-formulation studies, achieving an average particle size of 119.6 nm, a PDI of 0.248, and an encapsulation efficiency of 88%. *In vitro* release studies demonstrated an accelerated drug release profile, with nearly all the khellin being released within 2 hours, whereas the control gel released only approximately 60% of the drug over 24 hours. This rapid release profile aligns well with khellin's mechanism of action. The release kinetics followed the Korsmeyer-Peppas model, with a calculated 'n' value of 0.058, indicating a controlled release mechanism. *Ex* *vivo* skin deposition studies revealed that khellin in the nanogel formulation exhibited 1.7 times greater skin deposition compared to the control gel. These findings suggest that the nanogel formulation offers a promising approach for enhanced khellin delivery, potentially improving psoriasis treatment outcomes when combined with phototherapy. Further investigations were warranted to evaluate the formulation’s toxicity and therapeutic efficacy in cell culture models and clinical settings.

## AUTHORS’ CONTRIBUTIONS

The authors confirm contribution to the paper as follows: Carried out experiments: S. Topuzoğlu, L. Abdo and S. Gümrükçü; Study concept, conceptualism, writing, reviewing, editing: E.A. Yapar; Data analysis or interpretation: İ. Esentürk-Güzel and A.G. Yurtsever; Data Curation: M.S. Erdal; Writing - reviewing and editing: R.K. Sindhu. All authors reviewed the results and approved the final version of the manuscript.

## Figures and Tables

**Fig. (1):**
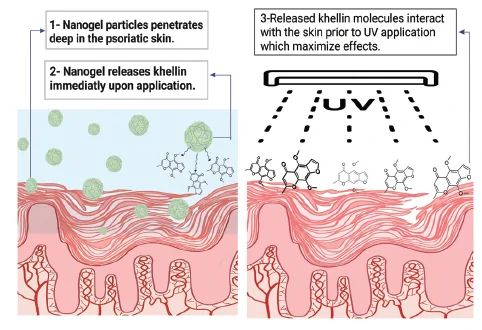
Mechanism of khellin-loaded nanogel in psoriasis.

**Fig. (2):**
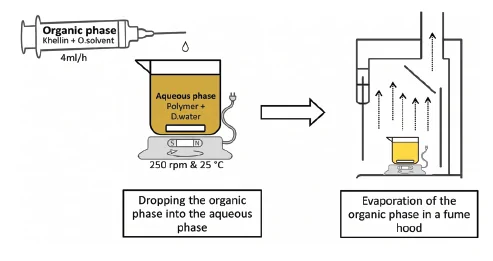
Preparation of the Nanogel.

**Fig. (3):**
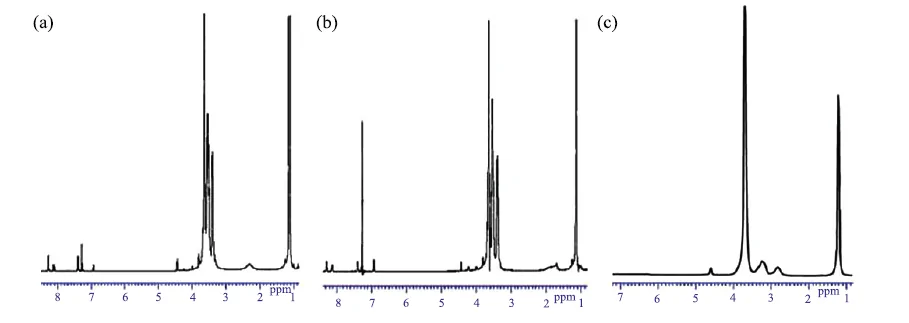
¹H-NMR spectra of the synthesized compounds: (**a**) NPC-P123-CH₃, (**b**) NPC-P123-OH, and (**c**) Gelatin-Pluronic copolymer.

**Fig. (4):**
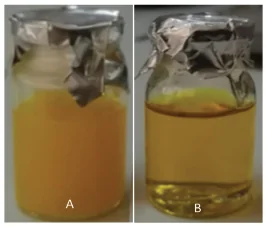
**A**) Nanogel prepared with 0.7% polymer. **B**) Nanogel prepared with 1.4% polymer.

**Fig. (5):**
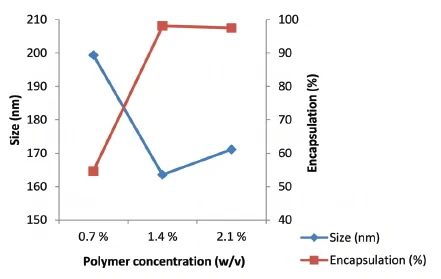
Comparison of the effects of polymer concentration on size and encapsulation efficiency.

**Fig. (6):**
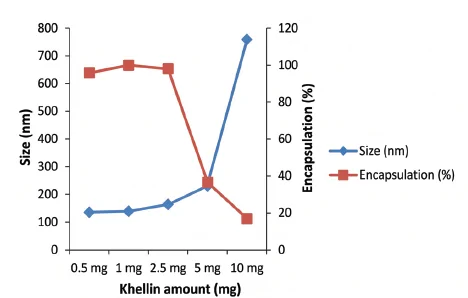
Comparison of the effects of the amount of active substance on size and encapsulation efficiency.

**Fig. (7):**
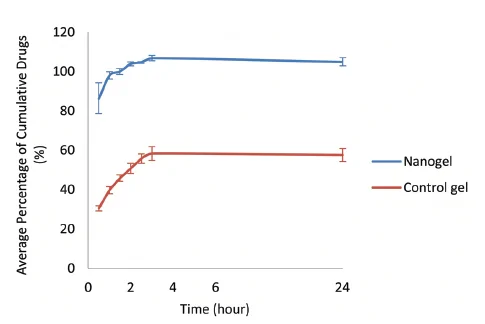
Graphs showing the mean cumulative drug percentage of nanogel and control gel versus time.

**Table 1:** Tested parameters during nanogel preformulation studies.

**Preformulation Parameters**	**Parameter Range**
Polymer concentration	0.7% (w/v) - 1.4% (w/v) - 2.1% (w/v)
Active ingredient amount	0.5 mg - 1 mg - 2.5 mg - 5 mg - 10 mg
Organic solvents	Acetone - Ethanol - Methanol
Evaporation methods of organic solvents	Heated rotary evaporator - Non-heated rotary evaporator - Fume hood
Additional applications	Sonication - Homogenization

**Table 2:** HPLC setup and operating conditions for khellin analysis.

**Parameter**	**Setting**
HPLC System	Shimadzu LC-10A
Pump	LC-20AT
Column Oven	CTO-10AS VP
Detector	DAD Detector
Column	C18, 250 mm × 4.6 mm, 5 µm
Temperature	35°C
Injection Volume	100 µL
Flow Rate	1 mL/min
Mobile Phase	3% Tetrahydrofuran, 47% Methanol, 50% Ultrapure Water
Linearity	r² = 0.9998 (0.0625–6.25 µg/mL)
Data Analysis	Microsoft Excel

**Table 3:** Effects of organic solvent types on PDI and size evaluated using dynamic light scattering spectrometry.

**Effects of Organic Solvents**	**Size (nm)**	**PDI**
Acetone	167 ± 2.931	0.240 ± 0.013
Methanol	184 ± 10.819	0.291 ± 0.049
Ethanol	261 ± 5.595	0.459 ± 0.076

**Table 4:** Comparison of heated and unheated fume hood and rotary evaporator methods effects on PDI and size of formulations with and without khellin.

**Fume Hood and Rotary Evaporator Effect**	**Size (nm)**	**PDI**
Heated rotary evaporator	142 ± 4.365	0.594 ± 0.071
Unheated rotary evaporator without khellin	176 ± 2.931	0.291 ± 0.013
Unheated fume hood without khellin	176.2 ± 0.306	0.306 ± 0.043
Unheated rotary evaporator with khellin	167 ± 2.931	0.240 ± 0.013
Unheated fume hood without khellin	163 ± 3.972	0.219 ± 0.003

**Table 5:** Homogenization effects on PDI and size of formulations.

**Fume Hood and Rotary Evaporator Effect**	**Size (nm)**	**PDI**
After homogenization	142 ± 4.365	0.594 ± 0.071
Before homogenization	167 ± 2.931	0.240 ± 0.013

**Table 6:** Effects of storage conditions on PDI and size of nanogel formulations.

**Time (weeks)**	**Size (nm)**		**PDI**	
	**25±2˚C / %60±5**	**2 - 8 °C**	**25±2˚C / %60±5**	**2 - 8 °C**
0	176 ± 1.850	176 ± 1.850	0.285 ± 0.003	0.285 ± 0.003
1	167 ± 1.582	185 ± 6.727	0.243 ± 0.014	0.269 ± 0.012
2	163 ± 1.124	180 ± 5.534	0.245 ± 0.011	0.279 ± 0.001
4	157 ± 1.320	171 ± 1.328	0.252 ± 0.008	0.297 ± 0.006
8	164 ± 2.107	180 ± 4.200	0.255 ± 0.001	0.297 ± 0.016
12	190.4 ± 2.991	176.6 ± 1.007	0.433 ± 0.029	0.236 ± 0.013
16	154.4 ± 0.709	169.6 ± 2.426	0.307 ± 0.009	0.248 ± 0.004
20	150.5 ± 3.904	167.5 ± 1.204	0.318 ± 0.007	0.259 ± 0.003

## Data Availability

The data and supportive information are available within the article.

## LIST OF ABBREVIATIONS

UVA: Ultraviolet A
UVB: Ultraviolet
KH: Khellin
GE: Gelatin
Ami: 3-amino-1-propanol
NMR: Nuclear Magnetic Resonance
HPLC: High-Performance Liquid Chromatography
PDI: Polydispersity Index
